# Comparisons of nonpharmaceutical analgesia and pharmaceutical analgesia on the labor analgesia effect of parturient women

**DOI:** 10.1002/iid3.869

**Published:** 2023-07-12

**Authors:** Rongyu Zhu, Qin Pan, Xiaoxia Cao

**Affiliations:** ^1^ Department of Anesthesiology Central Hospital of Enshi Tujia and Miao Autonomous Prefecture Enshi Hubei Province China

**Keywords:** epidural anesthesia, guide instrument, labor analgesia, nonpharmaceutical analgesia, pharmaceutical analgesia

## Abstract

**Objective:**

We aimed to compare the labor analgesia effects of nonpharmaceutical analgesia and pharmaceutical analgesia on parturient women.

**Methods:**

One hundred and four parturient women with spontaneous births were selected and randomly divided into pharmaceutical and nonpharmaceutical analgesia groups. Before and after analgesia, the Visual Analogue Scale (VAS), parturient satisfaction with analgesia, serum pain stress factors (substance P [SP], neuropeptide Y [NPY], nerve growth factor [NGF], and prostaglandin E2 [PGE2]), duration of labor, vaginal bleeding at 2 h postpartum, postpartum urinary retention and dysuria incidence, Apgar score of 1 min and 5 min after birth, and neonatal cord blood gas analysis (pH, partial pressure of oxygen [PO_2_], partial pressure of carbon dioxide [PCO_2_], and lactate [Lac]) were compared in the two groups.

**Results:**

VAS scores were lower and the analgesia satisfaction was higher in the pharmaceutical analgesia group than in the nonpharmaceutical analgesia group (all *p* < .05). Serum levels of SP, NPY, NGF, and PGE2 in the pharmaceutical analgesia group were lower than those in the nonpharmaceutical analgesia group (all *p* < .05). The first and second stages of labor were longer and the bleeding volume at 2 h postpartum was greater in the pharmaceutical analgesia group than those in the nonpharmaceutical analgesia group (all *p* < .05). Reduced Lac and PCO_2_ levels and increased PO_2_ level were found in the pharmaceutical analgesia group in comparison to the nonpharmaceutical analgesia group (all *p* < .05).

**Conclusion:**

This study demonstrates that the analgesic effect and neonatal condition of the pharmaceutical analgesia are better than the nonpharmaceutical analgesia, but the labor duration and postpartum bleeding volume of the pharmaceutical analgesia are greater than those of the nonpharmaceutical analgesia.

## Graphical abstract

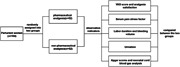

## 1 INTRODUCTION

Labor is one of the most significant painful experiences in women's lives, and the management of labor pain is a vital moment, not only to provide more comfort, but to also reduce stress and suffering.^1^, ^2^ Labor pain is a physiological phenomenon resulting from uterine contraction in the process of delivery, often accompanied with tension, anxiety, as well as a series of adverse emotions.^3^ The ideal labor analgesia technique should have a rapid onset, adjustable depth and duration, as well as predictable quality. Besides, it should also be easy to conduct and have minimal side effects to mother and fetus.^4^ Epidural analgesia is widely applied in the remission of labor pain, and intravenous opioid‐analgesics or nonmedical pain relief ways can offer an alternative in specific situations.^5^, ^6^ However, the management of labor pain remains an essential topic necessitating considerable attention due to the debilitating impacts of severe labor pains.

Labor pain management is both a key issue for future mothers and a tremendous challenge in modern medicine. There are currently many pharmacological and nonpharmacological labor pain relief approaches for pregnant women.^7^ Neuraxial anesthesia is defined as the most effective pharmacologic way for analgesia, and spinal, epidural, combined or not, as well as dural puncture epidural anesthesia are commonly techniques for neuraxial anesthesia.^8^ Epidural injection of amide anesthetics combined with opioids is commonly used to relieve labor pain due to the benefits of minimal dose and reduced side effects.^9^ Ropivacaine is widely used in obstetric anesthesia due to its good analgesic properties while without resulting in motor blockade or systemic toxicity.^10^, ^11^ Fentanyl is an appropriate analgesic drug in use for labor because of its low molecular weight and high potency.^12^ Some nonpharmacological pain management techniques include breathing techniques, water use, acupuncture/acupressure, massage, hypnotherapy, as well as transcutaneous electrical nerve stimulation (TENS) machine.^13^ TENS machine relieves pain by activating descending inhibitory systems of the central nervous system with a low‐voltage electrical current.^14^ Interestingly, TENS machine is often only available to women who have experienced a vaginal birth, while for women giving birth by cesarean, TENS machine may help improve birth satisfaction.^15^ In our research, we aimed to compare the labor analgesia effects of nonpharmaceutical analgesia and pharmaceutical analgesia (epidural anesthesia) on parturient women.

## 2 MATERIALS AND METHODS

### 2.1 Study subjects

A total of 104 primiparas admitted to Central Hospital of Enshi Tujia and Miao Autonomous Prefecture were enrolled as the study subjects, and the general conditions of the subjects are presented in Table 1.

**Table 1:** Comparison of the general conditions of women between the two groups.

General condition	Nonpharmaceutical analgesia group (*n* = 52)	Pharmaceutical analgesia group (*n* = 52)	*p* Value
Mean age (years)	28.41 ± 3.46	28.69 ± 3.75	.693
Gestational week (week)	38.47 ± 1.17	38.38 ± 1.39	.724
BMI (kg/m^2^)	26.53 ± 2.20	26.79 ± 2.81	.601
Smoking history (*n* (%))			.741
Yes	6 (11.54%)	4 (7.69%)	
No	46 (88.46%)	48 (92.31%)	
Abortion history (*n* (%))			.613
Yes	8 (15.38%)	11(21.15%)	
No	44 (84.62%)	41(78.85%)	

Abbreviation: BMI, body mass index.

Eligibility criteria: women with a gestational age of 37–42 weeks; women in active labor; gestation with a single fetus in the cephalic position; all women voluntarily received the labor analgesia method required by this study.

Exclusion criteria: women had contraindications to vaginal delivery such as abnormal bone and soft birth canal, placenta previa, placental abruption, multiple pregnancy, scarred uterus, and fetal malposition; women with pregnancy comorbidities; having a wound or inflammation in the skin areas where the TENS electrodes were applied; presence of a pacemaker.^16^

### 2.2 Research technique

After entering the active stage of labor, the participating women were randomly divided into pharmaceutical analgesia group (the analgesia was performed by epidural anesthesia during delivery, *n* = 52) and nonpharmaceutical analgesia group (*n* = 52, analgesia was performed using a delivery instrument muscle electrical stimulation).

Pharmaceutical analgesia group: the computer‐integrated patient‐controlled epidural analgesia (CIPCEA) studied by Sng et al.^17^ minimized the amount of anesthetics and improved maternal satisfaction. The parturient women were in a lateral lying position with flexion. The space between the second and the fourth lumbar vertebra was selected for epidural puncture and local anesthesia. The puncture was performed with a spoon‐shaped needle, and 5 mL 0.1% ropivacaine (Yichang Humanwell Pharmaceutical Co., Ltd; authorized document number: country medicine accurate character H20103636) was injected in the condition that no blood was drawn back after successful puncture, followed by injection of 5–7 mL of prepared solution (0.1% ropivacaine + 0.1 mg fentanyl (Yichang Humanwell Pharmaceutical Co., Ltd; authorized document number: country medicine accurate character H20054172) diluted to 80 mL) (the first dose). The plane was controlled at T10‐L3. After 20 min of the first dose, the PCEA pump was connected, and 10 mL was administered every hour. The bolus dose was 2 mL and the locking time was 20 min. If the analgesic effect was not good, the pregnant woman or the midwife could press the bolus button until the required analgesic effect has been achieved. The pump was stopped when the uterine orifice was fully open.

Nonpharmaceutical analgesia group: the LeBeiEr delivery analgesia instrument (Wuhan Runze Hongye Medical Technology Co., Ltd) was used for parturient women. Meanwhile, parturient women could also cooperate with the use of multifunctional birth‐stool, and parturient women could freely choose the body position. Before the use of LeBeiEr delivery analgesia instrument, the women should be informed the knowledge of this instrument, and the attention and feeling when using. Besides, it was necessary to inform parturient women that the analgesic instrument had no side effects on the mother and baby, and the operation methods for this analgesic instrument should be correctly guided. During the whole delivery process, the senior midwife could participate in and guide the parturient women in all stages of the delivery methods, so that the women could get emotional support, improve the sense of security, establish confidence in delivery, and ensure a smooth delivery by the way of verbal comfort, massage, and assist in balance. In the meantime, midwifes should closely monitor the progress of the maternal labor process to assist the parturient women to achieve their satisfactory analgesic effect.

### 2.3 Sample collection and processing

Blood samples: the peripheral fasting venous blood of the two groups was collected before and 2 h after analgesia for detection, and the harvested blood samples were centrifuged for serum collection.

Umbilical cord blood specimens: the two groups of newborns were routinely treated and promptly clamped with an umbilical cord of about 15 cm long. The umbilical cord extraction site was disinfected by iovolol, and about 1 mL of the umbilical artery blood was extracted with a heparin‐contained syringe (2.5 mL). After the extraction, the needle tip was immediately sealed with a rubber plug to avoid gasification and immediately sent for inspection. The special person was responsible for registering the sample information, and performed the umbilical cord blood gas analysis of newborns in the two groups with an automatic blood gas analyzer and biochemical instrument (ABL80, Radiometer Medical A/S).

### 2.4 Indicator observation

Visual analogue scale (VAS): VAS was utilized for evaluating the pain level before analgesia and at 30, 60, and 120 min after analgesia. The VAS was a simple one‐dimensional instrument used worldwide to assess pain intensity. It was characterized by a 10‐cm‐long horizontal line, with 0 representing no pain, and 10 representing the most severe pain. In clinical use, the side of the scale was carried back to the patient, and let the patient mark the corresponding position on the ruler. The participating women marked the position according to her subjective pain feeling, and the medical staff evaluated the score according to the position marked by the patient.^3^

Analgesia satisfaction: After delivery, the participating women were satisfied with the analgesia, with a full score of 10 points. Zero was not satisfaction, and 10 was the most satisfaction. The higher score represented the higher satisfaction.

Serum pain stress factors: before and after analgesia, the serum pain stress factors, including substance P (SP), neuropeptide Y (NPY), nerve growth factor (NGF), and prostaglandin E2 (PGE2) were detected by ELISA kit (mlbio).

Time of labor: the midwife recorded the first, second and third stage of labor of the woman respectively. The first stage of labor started from the onset of regular contractions of about 5–6 min to the time of full uterine opening. The second stage of labor was from the uterine cervix to the delivery of the fetus. The third stage of labor was from fetal delivery to the shedding of the placenta.

Vaginal bleeding volume: the vaginal bleeding volume at 2 h postpartum in both groups were observed and recorded.

Urination: the incidence of urinary retention and dysuria after delivery (delivery to 6 h postpartum). Urinary retention in this study meant that a large amount of urine could not be discharged, and this study included those who ultimately need a one‐time catheter or an indwelling catheter to relieve the bladder filling. Dysuria referred to laborious urination, waiting for urination, intermittent urination line, as well as weakness.

Apgar score: the Apgar score of 1 min and 5 min after birth was recorded, respectively. The Apgar score was assessed by five criteria: activity, pulse, reflex irritable face, skin color, and breathing. After delivery, the child was scored according to these criteria, and the sum of 5 values was the Apgar score.^18^

Blood gas analysis of umbilical cord blood in newborns: the hydrogen ion concentration (pH), oxygen partial pressure (PO_2_), carbon dioxide partial pressure (PCO_2_), and lactic acid (Lac) in two groups of newborns were compared. pH could directly reflect the pH of blood, and then made a preliminary judgment on whether it was an acid–base balance disorder. Because the timing of the specimen collection was before the fetus was crying, so the PO_2_ and PCO_2_ test results could directly reflect the gas exchange condition between the mother and the fetus, which was an important indicator to reflect the respiratory condition of the fetus. Lac could assess the anaerobic metabolism during fetal delivery.^19^

### 2.5 Statistical analysis

SPSS21.0 statistical software was used for statistical analysis. The measurement data, expressed with mean ± standard deviation, were compared by *t* test. Enumeration data, expressed in percentage or rate, were analyzed by Fisher's exact test. *p* < .05 indicated a significant difference.

## 3 RESULTS

### 3.1 Maternal general situation

The mean age, gestational week, body mass index (BMI), smoking history, and abortion history were not significantly different in women between the pharmaceutical analgesia and nonpharmaceutical analgesia groups (all *p* > .05; Table 1).

### 3.2 VAS score and analgesia satisfaction

Before analgesia, the VAS scores were not significantly different in women between the pharmaceutical analgesia and nonpharmaceutical analgesia groups (*p* > .05), while at 30, 60, and 120 min after analgesia, the VAS score was decreased when compared to that before analgesia (all *p* < .05). Additionally, there were reduced VAS score and elevated satisfaction level in women of the pharmaceutical analgesia group in contrast to the nonpharmaceutical analgesia group (both *p* < .05) (Table 2).

**Table 2:** Comparison of VAS scores and satisfaction of women before and after analgesia between the two groups.

		Nonpharmaceutical analgesia group (*n* = 52)	Pharmaceutical analgesia group (*n* = 52)
VAS scores	Before analgesia	8.15 ± 0.53	8.12 ± 0.61
	30 min after analgesia	5.46 ± 0.64*	4.36 ± 0.63* ^,^ **
	60 min after analgesia	4.84 ± 0.69*	3.52 ± 0.78* ^,^ **
	120 min after analgesia	4.58 ± 0.52*	2.94 ± 0.52* ^,^ **
Satisfaction		7.93 ± 0.57	8.88 ± 0.68**

Abbreviation: VIS, Visual Analogue Scale.

* *p* < .05 vs. before analgesia.

** *p* < .05 vs. nonpharmaceutical analgesia group.

### 3.3 Serum pain stress factor

Before analgesia, no difference was observed in the levels of serum pain stress factors (SP, NPY, NGF, and PGE2) in serum of women between the pharmaceutical analgesia and nonpharmaceutical analgesia groups (all *p* > .05), while the levels of these factors were decreased after analgesia in comparison to before analgesia (all *p* < .05). Besides, there exhibited reduced levels of serum pain stress factors in the pharmaceutical analgesia group compared to the nonpharmaceutical analgesia group (all *p* < .05) (Table 3).

**Table 3:** Comparison of serum pain stress factor levels of women before and after analgesia between the two groups.

		Nonpharmaceutical analgesia group (*n* = 52)	Pharmaceutical analgesia group (*n* = 52)
SP (μg/mL)	Before analgesia	7.36 ± 0.78	7.54 ± 0.74
	After analgesia	5.67 ± 0.66*	4.39 ± 0.57* ^,^ **
NPY (μg/mL)	Before analgesia	268.24 ± 29.91	265.48 ± 27.36
	After analgesia	241.65 ± 25.21*	217.69 ± 22.26* ^,^ **
NGF (pg/mL)	Before analgesia	96.47 ± 9.14	94.55 ± 8.17
	After analgesia	65.25 ± 6.10*	51.39 ± 5.68* ^,^ **
PGE2 (pg/mL)	Before analgesia	270.15 ± 29.29	271.68 ± 26.14
	After analgesia	252.69 ± 24.67*	237.65 ± 21.16* ^,^ **

* *p* < .05 vs. before analgesia.

** *p* < .05 vs. nonpharmaceutical analgesia group.

### 3.4 Labor duration and bleeding volume

The first and second stages of labor were longer and the bleeding volume at 2 h postpartum was greater in the pharmaceutical analgesia group than those in the nonpharmaceutical analgesia group (all *p* < .05). No statistical difference was witnessed in the third stage of labor between the two groups (*p* > .05) (Table 4).

**Table 4:** Comparison of labor duration and bleeding volume of women before and after analgesia between the two groups.

	Nonpharmaceutical analgesia group (*n* = 52)	Pharmaceutical analgesia group (*n* = 52)
First stage of labor (min)	443.72 ± 71.65	529.73 ± 74.95*
Second stage of labor (min)	38.54 ± 4.46	46.43 ± 4.99*
Third stage of labor (min)	13.15 ± 3.28	13.42 ± 3.57
Bleeding volume at 2 h postpartum (mL)	205.35 ± 50.36	294.84 ± 57.25*

* *p* < .05 vs. nonpharmaceutical analgesia group.

### 3.5 Urination

As presented in Table 5, no change was found in urinary retention and dysuria after delivery between the pharmaceutical analgesia and nonpharmaceutical analgesia groups (both *p* > .05).

**Table 5:** Comparison of postpartum urination in the parturient women of two groups (*n* (%)).

	Nonpharmaceutical analgesia group (*n* = 52)	Pharmaceutical analgesia group (*n* = 52)	*p* Value
Urinary retention (*n* (%))	5 (9.62%)	7 (13.46%)	0.760
Dysuria after delivery (*n* (%))	5 (9.62%)	8 (15.38%)	0.555

### 3.6 Apgar scores and neonatal cord blood gas analysis

The results in Table 6 revealed that no change was observed in the Apgar scores of 1 min and 5 min in neonatus of the pharmaceutical analgesia and non‐pharmaceutical analgesia groups (both *p* > .05). Reduced Lac and PCO_2_ levels, along with elevated PO_2_ levels were witnessed in the pharmaceutical analgesia group in comparison to the nonpharmaceutical analgesia group (all *p* < .05). Moreover, no statistical significance was found in pH between the two groups (*p* > .05).

**Table 6:** Comparison of Apgar scores and neonatal cord blood gas analysis between the two groups.

		Nonpharmaceutical analgesia group (*n* = 52)	Pharmaceutical analgesia group (*n* = 52)
Neonatal Apgar score	1 min	8.34 ± 0.62	8.38 ± 0.69
	5 min	9.21 ± 0.75	8.98 ± 0.96
Lac (mmol/L)		6.67 ± 1.46	2.64 ± 1.24*
PCO_2_ (mmHg)		58.64 ± 3.68	42.16 ± 3.29*
PO_2_ (mmHg)		23.86 ± 2.64	32.36 ± 2.69*
pH		7.18 ± 0.67	7.36 ± 0.68

* *p* < .05 vs. nonpharmaceutical analgesia group.

## 4 DISCUSSION

The pain relief in labor is regarded as a routine component of intrapartum care in many countries, and all pregnant women have access to their chosen method of pain relief.^20^ Currently, pain management strategies include pharmacological interventions (for relieving the labor pain) and nonpharmacological interventions (helping women cope with labor pain).^21^ The pharmacological interventions such as epidural analgesia are useful for pain management in labor but with side effects, while the nonpharmacological interventions have few adverse effects on both maternal and fetal outcomes.^22^ Therefore, a better recognition of labor pain is essential for developing strategies to help women develop capacity to reduce drug interventions.^23^ In our work, we designed to compare the labor analgesia effects of nonpharmaceutical analgesia and pharmaceutical analgesia (epidural anesthesia) on parturient women.

Globally, epidural analgesia, as the most important pharmacological intervention, is found to be an effective way of pain relief, but it is not necessarily related to a positive experience of birth.^24^ In our study, the combination of 0.1% ropivacaine and 0.1 mg fentanyl was used for epidural analgesia, and the findings suggested that the analgesic effect and neonatal conditions of the pharmaceutical analgesia were better, while the labor duration and postpartum bleeding volume of the pharmaceutical analgesia were greater. Basarinaite and colleagues have obtained the same findings, which reveal that women who are in the first stage of labor are more likely to select epidural analgesia to avoid the discomfort that may happen during delivery.^25^ Additionally, evidence has shown that the combination of low concentrations of local anesthetics and lipid‐soluble opioids do not hinder the labor progress or depress the newborn.^26^ Especially, the administration of both ropivacaine and sufentanil, as a common approach for labor analgesia, exerts significant effects on postoperative pain management.^27^, ^28^, ^29^ However, data from a prior article has demonstrated that women who select epidural analgesia exhibit an elevated risk of prolonged labor and cesarean delivery in comparison to those select other types of analgesia or even without analgesia.^30^

In contrast, various nonpharmacological therapies seem to be safe to minimize pain intensity, increase maternal satisfaction, as well as delay pharmacological analgesics.^13^, ^31^ Nonpharmacological approaches of pain relief may be utilized alone or with the multidisciplinary team (e.g., doctors, nursing technicians, and doulas), which depends on the choice of parturient women and the hospital's infrastructure.^16^ In our study, the LeBeiEr delivery analgesia instrument was used for nonpharmacological approaches of pain relief in pregnant women, with the findings revealed that the nonpharmaceutical analgesia showed shorten labor duration and less postpartum bleeding volume. As reported, TENS is safe for both mother and fetus, and plays an active role in the delivery process, such as reducing anxiety, pain, delivery duration, and the use of auxiliary analgesia, as well as improving satisfaction.^32^, ^33^ Besides, TENS therapy provides women with a low‐cost and low‐intervention approach to manage early labor, and creates a sense of control that helps women control the discomforts during delivery.^34^ Nevertheless, the majority of the nonpharmacological pain relief wars are noninvasive and potentially safe for mothers and infants, but their efficacy remains undefined because of the absence of high quality evidence.^21^ Therefore, significant recommendations concerning labor analgesia such as administration way, dosage, substances, and maintenance of labor analgesia, should be followed. Meanwhile, a clinical assessment for the analgesia's indications or contraindications should be performed.^35^

In summary, our work suggests that the analgesic effect and neonatal condition of the pharmaceutical analgesia are better than the nonpharmaceutical analgesia, but the labor duration and postpartum bleeding volume of the pharmaceutical analgesia are greater than those of the nonpharmaceutical analgesia. This study provides further advice on the clinical choice of appropriate maternal analgesia. Nurses should consider in practice that pregnant women have the right to make autonomous choices on labor pain management during delivery based on their comprehension of pain relief regimens. Furthermore, it is of great significant to educate women about the pain relief approaches in labor and to help them deal with childbirth‐related fears.

## AUTHOR CONTRIBUTIONS

Rongyu Zhu finished study design, Qin Pan finished experimental studies, Xiaoxia Cao finished data analysis, Rongyu Zhu, and Qin Pan finished manuscript editing. All authors read and approved the final manuscript.

## CONFLICT OF INTEREST STATEMENT

The authors declare no conflict of interest.

## ETHICS STATEMENT

The study was ratified by the ethics committee of Central Hospital of Enshi Tujia and Miao Autonomous Prefecture. Written consent was acquired from all participants.

## Data Availability

The original contributions presented in the study are included in the article/Supporting Information, further inquiries can be directed to the corresponding author.
